# Illegitimate Tasks and Employees’ Turnover Intention: A Serial Mediation Model

**DOI:** 10.3389/fpsyg.2021.739593

**Published:** 2021-10-28

**Authors:** Xiaoye Zeng, Yafu Huang, Shouying Zhao, Lianping Zeng

**Affiliations:** School of Psychology, Guizhou Normal University, Guiyang, China

**Keywords:** illegitimate tasks, effort–reward imbalance, work–family conflict, turnover intention, employees

## Abstract

In the historical and cultural context of developing countries, such as China, illegitimate tasks have become an important source of workplace pressure for employees. Guided by the framework of the stress-as-offense-to-self theory, we explored how illegitimate tasks increase turnover intention. A total of 474 employees from China effectively completed the online survey. The results showed a positive correlation between illegitimate tasks, effort–reward imbalance, work–family conflict, and turnover intention. Illegitimate tasks can affect intention to quit directly and through two indirect paths: the separate intermediary effect of work–family conflict and the continuous mediating role of effort–reward imbalance and work–family conflict. The results indicate that illegitimate tasks increase employees’ intention to quit through the role of effort–reward imbalance and work–family conflict. This study contributes to our understanding of the mechanisms underlying the relationship between illegitimate tasks and workers’ turnover intention in the context of Chinese history and culture. Additionally, the findings have implications for reducing attrition rate.

## Introduction

The sources of workplace stress for employees have long been a common topic of concern. These sources—including social pressure, workload, and performance pressure—appear from all aspects of employees’ lives (Dai et al., 2021), but the effect of illegitimate tasks at work is often overlooked. For example, Semmer et al. (2006) interviewed 159 employees and found that approximately one-third of the tasks on their list were considered illegitimate. Moreover, in developing countries such as China, illegitimate tasks are even more inevitable (Zhang et al., 2019). Therefore, exploring the consequence variables of illegitimate tasks can enrich the research into stressors and inspire workers’ occupational stress interventions and negative behavior management. Illegitimate tasks are defined as tasks that employees perceive ought not to be performed by them and are beyond their professional expectations (Semmer et al., 2010). Further, exploring this topic can provide a new perspective to explain employee stress and negative reactions as important factors affecting employees’ work behavior and physical and mental health. For example, illegitimate tasks can negatively affect their perceptions, work–family relationships, and work attitudes (Ahmed et al., 2018; Apostel et al., 2018; Meier and Semmer, 2018; Schulte-Braucks et al., 2019). Thus, this study considers the consequence variables of such tasks.

In recent years, high employee turnover has gradually become a major challenge for many companies in China. High turnover rates indicate a labor shortage, resulting in high recruitment, staffing, and training costs. As the most direct predictor of quitting behavior, turnover intention refers to individuals’ subjective willingness to change their jobs within a certain period (Sousa-Poza and Henneberger, 2004). However, how illegitimate tasks affect employees’ turnover intention has rarely been explored in previous studies. Van Schie et al. (2014) showed that illegitimate tasks are negatively related to intention to stay as a volunteer in non-profit organizations. However, non-profit and for-profit organizations are different in nature and have different motivations and processes for leaving and staying (Holtom et al., 2008). In this regard, follow-up studies further found that illegitimate tasks lead to higher turnover intentions among German IT workers (Apostel et al., 2018), PhDs and postdocs (Bramlage et al., 2021). However, the relationship between illegitimate tasks and high turnover may vary across socio-cultural contexts as well as across occupational groups. Therefore, this study further explores the relationship in a Chinese cultural context with a sample of employees from different occupations. In addition, we examined the internal mechanisms of the above relationship.

## Illegitimate Tasks and Turnover Intention

Illegitimate tasks violate employees’ reasonably expected behavioral norms (Meier and Semmer, 2018; Zhao et al., 2021). They have two core characteristics: they (1) are outside the scope of the self-role and (2) offend the individual’s professional identity. First, illegitimate tasks are often outside the employees’ role, thus violating and reflecting a gap in their role expectations. Workers may perceive this as discrimination and feel that their self-esteem is threatened (Eatough et al., 2016; Schulte-Braucks et al., 2019). The theory of stress-as-offense-to-self argues that individuals see threats to self-esteem as central to the experience of stress. Further, to sustain and defend their positive self-image, workers may take appropriate coping measures (e.g., leave their jobs, counterproductive work behavior; Semmer et al., 2015; Zhao et al., 2021). Empirical studies have shown that employees adopt exit strategies to avoid damaging their self-esteem (Baumeister, 1996; Lazanis, 1999). Second, illegitimate tasks offend employees’ professional identities and cause tension and stress, constituting a stressor that threatens their identity (Semmer et al., 2007; Stefanie et al., 2016; Schulte-Braucks et al., 2019). Individuals tend to make free choices about their actions based on full awareness of their personal needs and information about the environment in which they live (Van Schie et al., 2014). When the current work environment does not meet an individual’s identity needs, they may consider changing jobs. Thus, illegitimate tasks triggering an intention to quit may be a coping response by individuals to avoid these self-threatening stressors. Hence, on the strength of prior evidence, we formulated the following hypothesis:

*Hypothesis 1: Illegitimate tasks would positively be related to turnover intention.*

## Mediating Role of Effort–Reward Imbalance and Work–Family Conflict

Effort–reward imbalance means that jobs and tasks characterized by high effort (i.e., job requirements and obligations) and low reward (i.e., money, respect, and job/career opportunities) can lead to employee stress and impaired well-being (Siegrist, 1996). According to the effort–reward imbalance theory, people’s time and energy at work should be compensated through pay, respect, and development prospects (Xie et al., 2021). If organizations do not give workers these corresponding rewards, the latter will feel the effort–reward imbalance and change their work status (Luo et al., 2007). Accordingly, this study concludes that illegitimate tasks may cause a sense of effort–reward imbalance among workers and increase their turnover intention for the following reasons: (1) the assignment of illegitimate tasks indicates a company’s disrespect for its staff (Eatough et al., 2016). In this situation, the staff members feel they are unfairly treated (Munir et al., 2017; Ahmed et al., 2018) and believe their efforts are not rewarded accordingly, creating a sense of effort–reward imbalance (Omansky et al., 2016). (2) Previous research has indicated that the tendency to resign is a potential employee reaction when their current work is dominated by high requirements and low reciprocations (Schaufeli and Bakker, 2004). Furthermore, Derycke et al. (2010) found that this imbalance between high requirements and low reciprocations increased workers’ turnover intention, indicating that when workers perceive an effort–reward imbalance, they may resort to leaving to cope with their current imbalanced psychological state. This was verified in a study in which elementary school teachers’ burnout and turnover intention were examined (Loerbroks et al., 2013). Studies on health care workers also indicate that individuals with a greater sense of effort–reward imbalance have a higher intention to quit; that is, the effort–reward imbalance can positively predict turnover intention (Tremblay et al., 2008; Luo et al., 2011). Bramlage et al. (2021) found that illegitimate tasks could cause Ph.D. students to feel an effort–reward imbalance and contribute to their turnover intention. Thus, the following hypothesis is offered:

*Hypothesis 2: The relationship between illegitimate tasks and turnover intention would be mediated by effort–reward imbalance.*

Work–family conflict is an inter-role conflict caused by the pressure of playing a role at work and home, and it usually takes the three major forms of temporal-, pressure-, and act-based conflict (Boles et al., 1997). Kottwitz et al. (2012) found that illegitimate tasks increase workers’ cortisol levels, suggesting that illegitimate tasks, as role stressors in the work domain, stimulate stress responses in individuals, causing them to feel high levels of stress both psychologically and physiologically. According to the border and conservation-of-resource theories, when individuals perceive role pressure in a certain domain, they reallocate their resources among different roles and achieve cross-domain resource transfer (Jin et al., 2014; Matthews et al., 2014). However, the time and energy available to employees at a given time are limited. Thus, when employees do not have enough resources to complete tasks in the work domain, they tend to use resources (e.g., time or energy) originally allocated to the family domain. This leads to conflicts between the two work–family domains (Jin et al., 2014; Matthews et al., 2014), as demonstrated in empirical studies. For example, Meier and Semmer (2018) found that illegitimate tasks contribute to work–family conflict. Similarly, Ahmed et al. (2018) validated the positive impact of illegitimate tasks on work–family conflict through a lack of sense of interactional fairness and negative emotions in the workplace. Unnecessary work may lead to extra effort, which may result in overtime and work–family conflict (Wang and Ye, 2011). Unreasonable tasks may cause rumination on work issues even at non-work times, which may contribute to work–family conflict (Zhou et al., 2020). According to the attribution theory, when work conflicts with family life, people attribute such conflict to stressors from the workplace. Thus, they have thoughts of leaving (Allen et al., 2000), as has been supported by empirical research. Mansour and Tremblay (2016) showed that work–family conflict positively affects workers’ intention to leave through job stress and burnout factors. Aboobaker et al. (2017) showed that the job and family’s temporal- and pressure-based conflict had the highest correlation with employees’ turnover intention. Wu et al. (2018) showed that work–family conflict is notably and positively related to the turnover intention. Thus, we hypothesized the following:

*Hypothesis 3: The relationship between illegitimate tasks and turnover intention would be mediated by work–family conflict.*

According to the job demand-resource model, work features can be classified into two major groups: work demands (rewards) and work resources (rewards; Bakker and Geurts, 2004; Bakker and Demerouti, 2014; Jiang and He, 2020). Job requirements are the continuous physical or mental efforts required of the workers by the company. Job resources refer to various kinds of support and rewards the organizational environment or platform can give to employees. Workers experience work–family conflict when they have excessive work demands that are not matched equally by the resources, as these work demands trigger feelings of exhaustion that spill over into non-work areas (Bakker and Geurts, 2004). Increased work demands (i.e., ways of working that need effort and input) and lack of work resources foretell energy expenditure, damaged health, and work–family conflict, as shown in previous research. Gorgievski et al. (2018) showed that stress-based giving spills over into the work–family domain through a process of continuous depletion and that work endeavor is the most significant predictive factor of work–family conflict. Willis et al. (2008) showed that the degree of impaired pay and reward was a strong predictor of work–family interference. It follows that illegitimate tasks could create a sense of effort–reward imbalance, giving rise to work–family conflict and, in turn, increasing employees’ intention to quit. Thus, the following hypothesis is offered:

*Hypothesis 4: Illegitimate tasks would affect turnover intention through a series of mediating effects of effort–reward imbalance and work–family conflict.*

**FIGURE 1:**
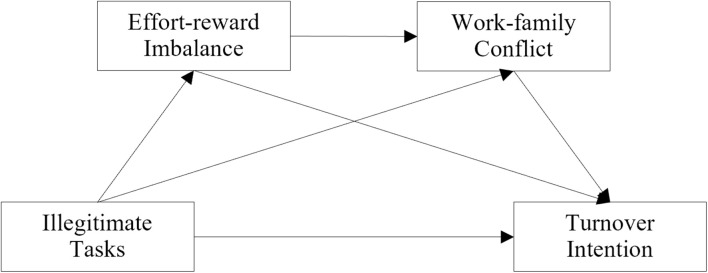
Research model.

In summary, this research explores the positive influence of illegitimate tasks on workers’ intention to resign based on the stress-as-offense-to-self theory, effort–reward imbalance model, and dual-process model of work–family interference. Further, this study explores the mechanisms underlying the influence of illegitimate tasks on intention to quit, namely, the mediating role of effort–reward imbalance and work–family conflict. Finally, this study provides a theoretical reference for reducing employees’ turnover intention. Figure 1 depicts the study model.

## Materials and Methods

### Participants and Procedure

Given the study’s constraints, we used an online questionnaire platform powered by www.wjx.cn to collect data. A convenience sampling method was used. To increase the response rate, we selected a short questionnaire format to assess the study variables. Participants could only submit questionnaires after all items were completed to decrease the probability of unexpectedly missing items. Depending on the logged-in WeChat account, a participant could submit only one response. Since self-report questionnaires may be subject to participant response deviation, all participants retained their anonymity and volunteered to participate. Furthermore, in the instructions for completing the scale, participants were informed that “This study is purely a scientific investigation and is not related to your work. There are no good or bad answers, but by analyzing the data, we can tell if you are not providing honest/objective responses.” Participants were also informed that they could discontinue midway if they felt uncomfortable. Due to the segregation policies and mobility restrictions, questionnaires were sent to employees across China *via* WeChat and email. Complete surveys were excluded if all items had the same answer, or marked responses had a clear pattern, the results were contradictory, or the response time was less than 15 min or more than 20 min. A total of 513 questionnaires were distributed, and after eliminating invalid questionnaires, 474 valid questionnaires were obtained. The effective participation rate was 92.398%. Table 1 presents the specific sample distribution conditions.

**TABLE 1:**
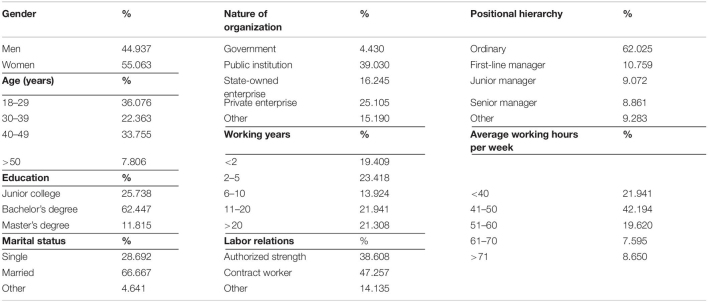
Sample distribution.

### Measures

Illegitimate tasks were measured using the Illegitimate Tasks Scale developed by Semmer et al. (2010), translated into Chinese by Zhang et al. (2019). The Chinese version has shown good reliability in measuring illegitimate tasks among Chinese workers (e.g., Liu et al., 2019; Yan et al., 2021). The questionnaire has eight items across two dimensions: unreasonable tasks (e.g., Do you have work tasks to take care of, which you believe should be done by someone else?) and unnecessary tasks (e.g., Do you have work tasks to take care of, which keep you wondering if they would not exist?). Items were rated on a 5-point Likert scale (1: strongly disapprove, 5: strongly approve). The average score of the eight items was calculated, with higher scores indicating that more illegitimate tasks were undertaken. The internal consistency for this questionnaire was α = 0.819.

Effort–reward imbalance was evaluated using the Chinese version of the Effort–Reward Imbalance Questionnaire developed by Siegrist et al. (2004) translated by Li et al. (2006) to assess the degree of the sense of effort–reward imbalance. The questionnaire totaled 23 items across three dimensions: effort (e.g., I have a lot of responsibility in my job), reward (e.g., I receive the respect I deserve from my superiors), and overcommitment (e.g., I get easily overwhelmed by time pressures at work). The effort and reward subscales were chosen as this study did not consider overcommitment. The Chinese version of the scale shows good reliability in measuring Chinese workers’ effort–reward imbalance (e.g., Yang and You, 2017; Fang et al., 2018). All 17 questions were rated on a 5-point Likert scale (1: quite disapprove, 5: quite approve). For each project, employees were asked to choose how these representations correspond to their reality.


Effort–reward ratio=effort/(reward×0.5454)

The effort–reward ratio converging to zero indicates less effort and higher reward. A payoff ratio over 1 indicates that the employee expends much effort but is not rewarded accordingly. The internal consistency for this questionnaire was α = 0.696.

Work–family conflict was evaluated using the Work–Family Conflict Dimension developed by Grzywacz and Marks (2000) and revised by Zeng and Yan (2013). The Chinese version of this subscale showed good reliability in measuring work–family conflict among Chinese employees (e.g., Zeng et al., 2021). The scale has four items (e.g., Stress at work makes you irritable at home.), rated on a 5-point Likert scale (1: completely disagree, 6: completely agree). The average score of the four items was calculated, and higher scores indicated stronger work–family conflict. The internal consistency for this questionnaire was α = 0.898.

Turnover intention was assessed using the Turnover Intention Scale developed by Mobley et al. (1978). Further, the Chinese version has shown good reliability in measuring Chinese workers’ intention to quit (e.g., He et al., 2020; Li et al., 2020). The scale has four items (e.g., I often want to quit my current job), rated on a 5-point Likert scale (1: completely disagree, 5: completely agree). The average score of the four items was calculated; higher scores indicated stronger turnover intention. The internal consistency for this questionnaire was α = 0.912.

The results of previous studies have suggested there are gender differences in illegitimate tasks (Omansky et al., 2016). Men reacted more than women to illegitimate tasks through the mechanism of perceived effort–reward imbalance. Since significant gender differences exist across the work situations for different occupations in China, gender was controlled for in the data analysis.

### Data Analysis

Common methodological bias was tested using SPSS 25.0 for Harman’s one-way test. Additionally, SPSS 25.0 was used for multicollinearity tests, fail-safe analysis, and Pearson’s correlations. The hypotheses were tested using the PROCESS macro in SPSS (modeling 6, 5,000 bootstrap resamples; Hayes, 2013).

## Results

### Multicollinearity Test and Common-Method Bias Test

Prior to data analysis, the raw data were pre-processed. First, a multicollinearity test was performed, and the tolerances ranged from 0.752 to 0.813 for each variable (all bigger than 0.1). Then, the variance expansion factors ranged from 1.230 to 1.329 (all smaller than 10). These results indicate that there was no significant multicollinearity between the variables.

Harman’s single-factor analysis was used to check for the presence of common methodological bias (Eby and Dobbins, 1997). The results indicated that there were seven factors with eigenvalues of more than 1, in which the first one interpreted 23.427% of the variability. This result is below the threshold of 40%, suggesting that there was not a serious problem with common-method bias in this study.

### Correlations Between Variables

Pearson’s correlation coefficients presented in Table 2 showed significant positive correlations between illegitimate tasks, effort–reward imbalance, work–family conflict, and turnover intention. Moreover, the correlation coefficients were moderate (far below Cronbach’s alpha coefficient), satisfying the requirements for hypothesis testing.

**TABLE 2:** Means, standard deviations, correlations, and reliabilities.

	*M*	*SD*	1	2	3	4	5
(1) Gender	1.450	0.498					
(2) IT	3.216	0.838	–0.084	**0.819**			
(3) ERI	3.332	0.455	–0.042	0.373***	**0.696**		
(4) WFC	2.914	1.020	–0.032	0.362***	0.442***	**0.898**	
(5) TI	2.744	1.107	0.031	0.236***	0.091*	0.206***	**0.912**

*p < 0.05, **p < 0.01, ***p < 0.001, N = 474 Chinese employees. IT, illegitimate tasks; ERI, effort–reward imbalance; WFC, work–family conflict; TI, turnover intention. The numbers in bold on the diagonal line are Cronbach’s alpha values.

### Hypothesis Testing

A serial mediation model was constructed in which gender was a control variable, illegitimate tasks an independent variable, turnover intention a dependent variable, and effort–reward imbalance and work–family conflict as Mediating Variable 1 and Mediating Variable 2, respectively.

**FIGURE 2:**
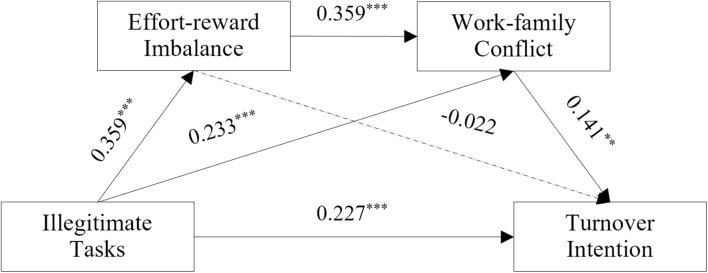
Roadmap of the influence of illegitimate tasks on turnover intention. *p < 0.05, **p < 0.01, ***p < 0.001.

Table 3 shows that illegitimate tasks were significant positive and valid predictors of quitting intention (β = 0.227, *p* < 0.001). In the path of “illegitimate tasks → effort–reward imbalance → turnover intention,” illegitimate tasks significantly and positively predicted effort–reward imbalance (β = 0.359, *p* < 0.001), and there was no significant effect of effort–reward imbalance on turnover intention (β = −0.022, *p* > 0.05). Thus, Hypothesis 3 was not verified (see Figure 2). In the path of “illegitimate tasks → work–family conflict → turnover intention,” illegitimate tasks significantly and positively predicted work–family conflict (β = 0.233, *p* < 0.001), and work–family conflict had a significant, positive effect on turnover intention (β = 0.141, *p* < 0.01). Thus, illegitimate tasks increased turnover intention by increasing work–family conflict. In the path of “illegitimate tasks → effort–reward imbalance → work–family conflict → turnover intention,” effort–reward imbalance positively predicted work–family conflict (β = 0.359, *p* < 0.001), suggesting that effort–reward imbalance and work–family conflict are highly correlated. Furthermore, illegitimate tasks increased employees’ work–family conflict by triggering a sense of effort–reward imbalance, thereby increasing the intention to quit, supporting Hypotheses 1, 2, and 4.

**TABLE 3:** Model results.

Variables	Model 1			Model 2			Model 3		
	ERI			WFC			TI		
	β	*SE*	*t*	β	*SE*	*t*	β	*SE*	*t*
Constant	0.122	0.251	0.487	–0.254	0.238	–1.067	–1.319	0.254	−5.189***
Gender	–0.042	0.086	–0.491	0.004	0.081	0.054	0.117	0.087	0.353
IT	0.359	0.043	8.398***	0.233	0.044	5.333***	0.227	0.048	4.738***
ERI				0.359	0.044	8.178***	–0.022	0.050	–0.449
WFC							0.141	0.049	2.870**
R^2^		0.159			0.243			0.143	
F		22.224***			30.011***			12.975***	

*p < 0.05, **p < 0.01, ***p < 0.001, N = 474 Chinese employees. IT, illegitimate tasks; ERI, effort–reward imbalance; WFC, work–family conflict; TI, turnover intention.

In these analyses, we observed the mediating effects of effort–reward imbalance and work–family conflict on the relationship between illegitimate tasks and turnover intention. Table 4 shows that the direct effect of turnover intention was 0.227. The effect size was 82.246%. The total indirect effect of effort–reward imbalance and work–family conflict was 0.049. The effect size was 17.754%. This shows a significant mediating role in the relationship between illegitimate tasks and turnover intention. Specifically, the mediating effect consisted of the indirect effects from two pathways. (1) The mediating effect of work–family conflict was 0.030 (95% CI: 0.008–0.066). The effect size was 10.870%. (2) The series of mediating effects of effort–reward imbalance and work–family conflict was 0.017(95% CI: 0.004–0.036). The effect size is 6.159%. Both indirect effect paths were significant. Further, the largest mediating effect path was through “illegitimate tasks → work–family conflict → turnover intention.” The results comparing the two mediating paths suggested that illegitimate tasks increased intention to leave primarily by increasing work–family conflict (see Table 4).

**TABLE 4:** Effects and 95% confidence intervals for Model 3.

	Effect	Bootstrap SE	Boot LLCI	Boot ULCI
Total effect	0.276			
Direct effects	0.227	0.048	0.133	0.321
Total indirect effect	0.049	0.023	0.004	0.097
Indirect effect 1	0.002	0.021	–0.040	0.041
Indirect effect 2	0.030	0.014	0.008	0.066
Indirect effect 3	0.017	0.008	0.004	0.036

*The path of indirect effect 1 is “illegitimate tasks → effort–reward imbalance → turnover intention,” the path of indirect effect 2 is “illegitimate tasks → work–family conflict → turnover intention,” and the path of indirect effect 3 is “illegitimate tasks → effort–reward imbalance → work–family conflict → turnover intention.”*

## Discussion

The current study investigated associations between illegitimate tasks and intent to leave for workers in China. Furthermore, the mediating effects of effort–reward imbalance and work–family conflict in these associations were investigated. Our findings show that illegitimate tasks have an indirect and positive effect on turnover intention. Additionally, the correlation between illegitimate tasks and intention to leave was mediated by work–family conflict. Further, this correlation was serially mediated by effort–reward imbalance and work–family conflict. Employees with illegitimate tasks were more likely to report higher effort–reward imbalance, further increasing their work–family conflict and, subsequently, increasing their intention to quit.

### Theoretical Implications

In China’s historical and cultural context, this study found a direct and positive effect of illegitimate tasks on workers’ intention to leave. Based on the perspectives of stress-as-offense-to-self and self-determination theory, we also demonstrated the effects of illegitimate tasks to answer the theoretical question of whether illegitimate tasks affect turnover intention in different occupational groups under the Chinese cultural background. We found a positive correlation between illegitimate tasks and turnover intention, consistent with the results of previous studies (Van Schie et al., 2014; Apostel et al., 2018; Bramlage et al., 2021). Thus, illegitimate tasks influence the intention to quit in developing countries as well as other contexts.

There are several possible explanations for the above findings. First, as stressors outside the scope of job roles, illegitimate tasks can prevent workers from performing core duties within the scope of the role. This deviates from their role expectations and sends a message of disrespect and lack of appreciation (Sonnentag and Lischetzke, 2017). Thus, employees perceive their professional identity as threatened and feel offended, leading to an increased turnover intention. Second, in accordance with the stress-as-offense-to-self theory, the influence of illegitimate tasks on turnover intention can be interpreted by individuals’ efforts to sustain a favorable self-image (Semmer et al., 2007, 2015). In other words, individuals tend to respond by leaving their jobs to avoid the adverse effects of illegitimate tasks on their favorable self-image. Third, when employees encounter illegitimate tasks, their work autonomy is hindered, and their needs are not met, increasing their willingness to leave (Van Schie et al., 2014). Finally, the completion of illegitimate tasks requires constant mental and emotional efforts, which can cause workers to develop health problems such as anxiety, depression, irritability, emotional exhaustion, and burnout (Semmer et al., 2015; Eatough et al., 2016; Munir et al., 2017; Meier and Semmer, 2018; Fila and Eatough, 2019). To maintain their own physical and mental health, employees’ turnover intentions increase. This is viewed as a cognitive coping style to avoid future re-exposure to such stress. This research provides empirical evidence to support the stress-as-offense-to-self theory and further validate the positive influences of illegitimate tasks on intention to quit.

There was no significant separate mediating effect of the effort–reward imbalance. There may be several reasons for this: (1) employees have no other option, as the labor market is limited and numerous conditions hinder employee mobility. (2) Employees themselves may tolerate the current imbalance for career strategy reasons (e.g., increased opportunities for career advancement). (3) In the global economic context of precarious employment, job insecurity, short-duration contracts, and intense wage competition, the problem of effort–reward imbalance is relatively small compared to the improved survival of employees.

This study identified the mediating effect of work–family conflict in the relationship between illegitimate tasks and turnover intention and found that effort–reward imbalance and work–family conflict had serial mediating roles in the relationship between illegitimate tasks and turnover intention. The findings support the effort–reward imbalance and the job demand-resource model, revealing the mechanisms underlying the association between illegitimate tasks and intention to quit in organizations. These findings further complement the literature on the psychological activity of individuals performing illegitimate tasks. The latent reasons for these results are as follows. In companies, illegitimate tasks are outside of the core elements of work, and their completion consumes the workers’ physical and mental resources. Therefore, it does not result in them receiving reciprocal work resources (Semmer et al., 2007). This results in the sense of effort–reward imbalance.

Negative emotions are triggered when employees experience an imbalance from high demand and low resources (Bakker et al., 2003). The accumulation of these emotions can trigger physical and psychological health issues, such as job burnout and emotional exhaustion (Semmer et al., 2015; Fila and Eatough, 2019), which can spill over into the family domain and lead to work–family conflict. According to the cognitive theory of emotion and expectancy theory of motivation, people do not exist passively in an imbalanced scenario of high effort and low gain. They try to reduce their efforts cognitively and behaviorally or maximize the reward and get back into a state of balance. In this case, individuals try to restore balance by leaving the job (Schmitt et al., 2015; Omansky et al., 2016). Based on the conservation of resources theory, the association between work–family conflict and the turnover intention can be represented as an inherent change process caused by the transfer of resources. Before resource allocation, individuals allocate resources according to the needs of different roles to balance the resources invested with the expected returns. Illegitimate tasks can increase individual role demands in the work domain and decrease their resources in the home domain, thus creating work–family conflict. This conflict increases when the return on resources in the work domain is lower than an individual’s expectations. Then, they are motivated to protect their own resources and minimize risks, resulting in withdrawal from resource inputs, characterized by an increased turnover intention (Zhang et al., 2016).

In sum, illegitimate tasks can create a sense of effort–reward imbalance. Moreover, the resulting emotions can spill over into the family sphere, creating work–family conflict, thus increasing employees’ willingness to resign. Thus, this series of mediating models effectively explained the influence of illegitimate tasks on turnover intention and the mediating pathways.

This study examined the association between illegitimate tasks and turnover intention in the context of China’s history and culture, using employees in different occupations as the study participants. Previous research mainly explored the role of illegitimate tasks on intention to quit for specific groups (e.g., information technology employees, volunteers, doctoral and postdoctoral) in other countries (e.g., Germany; Van Schie et al., 2014; Apostel et al., 2018; Bramlage et al., 2021). However, the relationship between the two in the Chinese cultural context was yet to be explored in depth. This study took this as an entry point. The reasons are as follows. First, as a member of a developing country, Chinese employees have a high benchmark of working hours, short paid vacation time, and more common overtime (Qian and Xu, 2019). This means that illegitimate tasks may be more prevalent in China compared to other countries (e.g., Germany). Second, Chinese culture emphasizes collectivism, high power-distance orientation, and cultural closeness (Wang et al., 2018). Collectivism focuses on collective values and collective interests (Wan and Zhang, 2021). Firms with high power-distance-oriented cultures focus on hierarchy and leadership authority (Zou and Chen, 2020). Cultural closeness means that people are punished or sanctioned when they deviate from these rules (Yang et al., 2020). In other words, employees are likely to be assigned illegitimate tasks under the condition of collective interest or under the orders of their leaders. Moreover, employees often choose to perform illegitimate tasks in order to avoid the penalties that come with disobeying orders. This is likely to result in overtime, which can lead to pay-reward imbalances and work–family conflicts. All of these can result in an intention to leave. The present study thus enriches the previous literature and provides a foundation for future research into illegitimate tasks in different groups and different historical and cultural contexts, especially those in developing countries worldwide. Furthermore, the negative results of illegitimate tasks in national development support the findings of previous studies. Accordingly, this study provides a theoretical reference for organizational interventions in developing countries.

### Practical Implications

In the current historical context of China, the economy has changed from high-speed growth to that of high-quality development. As builders of the nation and society, employees may find it difficult to avoid encountering illegitimate tasks in this context.

Such illegitimate tasks can have adverse consequences on workers, especially on their intention to quit. Therefore, organizations need to minimize the assignment of illegitimate tasks to address this problem. First, the perceived sensitivity of employees to illegitimate tasks can be reduced by increasing their power-distance orientation. Second, the perception of illegitimate tasks can be improved by expanding their job roles’ range of tasks and goals by increasing the roles’ flexibility. Finally, leaders can place illegitimate tasks in meaningful work situations by highly recognizing and praising the work done by employees.

Companies should strive to reduce the intensity of effort–reward imbalance in employees. First, leaders should adhere to the concept of “fairness first,” effective motivation, and people-oriented management. “Fairness first” means that the system development and management process should be based on the principle of prioritizing fairness. This can reduce the sense of imbalance among workers by reducing their horizontal comparison. Second, effective motivation involves enhancing career rewards by designing a scientific incentive system that gives each employee appropriate rewards and compensation (e.g., allowances, benefits, and improved working conditions). Finally, people-oriented management is a leadership practice to ensure the development and meet the needs of workers as human beings. Further, employers should provide them with spiritual support through “soft rewards” (e.g., professional development opportunities, professional honor, and cultural life), to reduce their sense of imbalance.

Organizations can reduce work–family conflict through leadership support and family care policies. Leadership support includes adopting a flexible work system, allowing employees to handle non-work matters during working hours, showing concern and understanding to take time off for family matters. A company’s family care policy involves providing social support to a worker’s children or parents. This in turn can enhance job satisfaction. Leadership support effectively avoids work–family conflict for employees, enhances their organizational commitment, and increases the utilization of family care policies.

## Limitations and Future Research

Future research could be conducted in the following areas. First, as this was a cross-sectional study, it could not infer the causal relationship between variables, and longitudinal studies using cross-lagged models need to be conducted in future. Second, it failed to explore family–work conflict, and future research could explore the effects of the two-way role of work and family. Finally, the percentage explained by indirect effects is small (17.754%). This may be due to the excessive occupational differences among participants, or it may be because most of the participants in this study worked for the government, public institutions, and state-owned enterprises. In China, jobs in these three types of organizations are not easily available, and labor rights are relatively secure. Future research could compare the differences between employees in different occupations.

## Conclusion

The study highlights the effects of illegitimate tasks in increasing employee’s turnover intention from the perspectives of the stress-as-offense-to-self theory, the effort–reward imbalance model, and the job demand-resource model. In addition, the results show that in the face of big growth and change, illegitimate tasks can cause an effort–reward imbalance in workers, increase their work–family conflict, and increase their intention to quit. It is hoped that this research will inspire further studies and debates on illegitimate tasks and workers’ turnover intention.

## Data Availability Statement

The raw data supporting the conclusions of this article will be made available by the authors, without undue reservation.

## Author Contributions

XZ and YH were in charge of literature review, data collection, data analysis, and writing. LZ and SZ supervised the topic selection and research design. All authors contributed to the article and approved the submitted version.

## Author Disclaimer

The views expressed are those of the authors.

## Conflict of Interest

The authors declare that the research was conducted in the absence of any commercial or financial relationships that could be construed as a potential conflict of interest.

## Publisher’s Note

All claims expressed in this article are solely those of the authors and do not necessarily represent those of their affiliated organizations, or those of the publisher, the editors and the reviewers. Any product that may be evaluated in this article, or claim that may be made by its manufacturer, is not guaranteed or endorsed by the publisher.
